# Resorption of a Fatty Tumor

**Published:** 1872-12

**Authors:** Jno. E. Lockridge

**Affiliations:** Augusta County, Virginia


THE GEORGIA

### MEDICAL COMPANION:

A MONTHLY ADVISER.

VOL. 2. ATLANTA, GA., DECEMBER, 1872. NO. 12.

### PART I.

### Original Communications and Special Selections.

### RESORPTION OF A FATTY TUMOR.

BY JNO. E. LOCKRIDGE, M. D., OF AUGUSTA COUNTY, VIRGINIA.

On April 26th, 1870, Miss S. B——, consulted me at my office, in regard to a tumor that had been gradually forming on the sub-maxillary region for the preceding ten or twelve years. For quite a number of years it was regarded by herself and friends as merely a so-called "double-chin."

The patient's age is about thirty, weight about 120; in figure, tall and spare; no disposition to obesity; her previous health has been uninterruptedly good. For the last two or three years the tumor has been growing rather rapidly, until at this date (April 26th, 1870), it constitutes a great deformity. In size and shape it very much resembles a tea-cup, inverted, as it were, upon the inframaxillary region, and extending from within half an inch of the point of the chin to the hyoid bone behind. The tumor was pendulous, perfectly free from pain or soreness on handling, and always had been; it imparted to the fingers the well-known elastic, fatty feel of fatty tumors. From the history, position and feel of the tumor, I had no hesitancy in pronouncing the growth to be the fatty variety; and I had but little hesitancy in giving the stereotyped opinion that "it is in vain to attempt the discussion of such tumors;" and that excission is the only remedy.

I remembered, however, that Sir Benjamin Brodie entertained somewhat a different opinion in regard to the discussion of such tumors. He was in the habit of effecting the resorption of such growths by the administration of liq. potassæ, as well as urgently recommending this treatment to others.

In as much as surgical operations are very much dreaded by all ladies, and in addition there being more or less danger attending the administration of chloroform, we agreed to postpone the operation for several months, and in the meantime try the efficacy of liq. potassæ, and ung. of iodine. Accordingly I furnished her with oz. iss. liq. potassæ and oz. i. ung. iodine, and directed her to take 25 drops of the former three times a day in a wine-glassful of sweetened water; and to rub into the tumor a portion of the ung. every night and morning; and to attend to it very faithfully and diligently. She being exceedingly desirous of getting clear of the great deformity, on the one hand, and the dread of having to undergo an operation on the other, it is needless to say the remedies were applied with great care and assiduity.

I saw her again in four or five weeks, and was very much pleased to find that the tumor was reduced in size about one-third. Another supply of the same medicine, in similar amount, was furnished the patient, and under its use the tumor was entirely absorbed in less than three months from the beginning of the treatment. It seemed that the region that a few months before was occupied by a large and unsightly tumor, now presented an appearance almost amounting to a concavity, so completely had the work been done; and what had been regarded as a 'double chin,' almost from childhood, had been, really, a constantly increasing tumor and deformity.

I have seen this patient within the last month, and there has not been the slightest return of the growth. The patient has been married since the cure and is the mother of one child.

I report this case to show that at least one more fatty tumor has been removed by absorption, a result which the most of surgeons regard as unattainable in any case; and as to the possibility of which I had been very sceptical myself, in as much as I have been compelled to remove by the knife such tumors, that had resisted all treatment having absorption in view.

I do not pretend to say the removal of this growth was due to the efficacy of either one of these remedies in particular, but I am rather inclined to the opinion that both the agents employed contributed a share to the highly successful and gratifying result.

And why should we not expect to effect the removal of fatty tumors by absorption? We know that nature chooses to convert some adventitious structures or exudations into fat for the very purpose of absorption. Take, for instance, the involution of the uterus after parturition. Before returning to its non-gravid size, the parietes undergo fatty degeneration and absorption.

Another instance we see in pneumonia; the products of inflammation, whether in the state of red or gray hepatization, frequently undergo fatty metamorphosis and absorption.

In what way did the administration of liq. potassæ and the local application of ung. iodine effect the removal of this fatty tumor? I think this question can be answered with at least a tolerable degree of physical and philosophical reasoning. I believe it is pretty well understood that liq. potassæ, or all alkalies, is a thinner of the blood; in fact, liquefiers of all the secretions. The contents of the vessels, whether blood-vessels or lymphatics, are then in the most favorable condition to absorb extraneous substances, either by directly absorbing them, or these liquefied contents of vessels may suffer diminution in quantity by increased exhalation, and thereby promote absorption according to the time-honored rule that the more empty the vessels the more active will be the absorption. Hence we see that both the vessels and their contents are placed in the most favorable condition for absorption by the action of the potassa.

And now for the agency of the iodine. I take it for granted that every physician admits of the absorbent power of iodine in the glandular system at least; as for instance, in the thyroid and mammary glands. I am just as well convinced of its absorptive powers over the blood-vessels and lymphatics. I have too often seen an enlarged liver or a pneumonic block, or a pleuritic effusion melt away under its use, even applied externally, to doubt for one moment its great and speedy power. If now, we combine these two very potent agents, potash and iodine, and add a third, the pressure exerted in faithfully rubbing in the ung., and thereby bringing the material to be absorbed forcibly in contact with the same vessels that voluntarily deposited the fat, or serum, or liquor sanguinis, as the case may be, I can see no good reason why we should not expect to effect the resoption of every fatty tumor that we meet with. Perhaps due time and diligence have not been taken in a great many cases.

I have purposely avoided even alluding to the saponifying theory of removing fatty formations, when speaking of the effects of liq. potassæ, for I conceive that the *modus operandi* of their absorption is sufficiently well explained without the necessity of resorting to such a fine-spun theory.

Mt. Solon, Virginia, November 14th, 1872.

